# Immunization with a Prefusion SARS-CoV-2 Spike Protein Vaccine (RBMRNA-176) Protects against Viral Challenge in Mice and Nonhuman Primates

**DOI:** 10.3390/vaccines10101698

**Published:** 2022-10-11

**Authors:** Qinhai Ma, Runfeng Li, Jianmin Guo, Man Li, Lin Ma, Jun Dai, Yongxia Shi, Jinlong Dai, Yuankeng Huang, Cailing Dai, Weiqi Pan, Huiling Zhong, Hong Zhang, Jian Wen, Haoting Zhao, Linping Wu, Wei Yang, Biliang Zhang, Zifeng Yang

**Affiliations:** 1 State Key Laboratory of Respiratory Disease, National Clinical Research Center for Respiratory Disease, Guangzhou Institute of Respiratory Health, The First Affiliated Hospital of Guangzhou Medical University, Guangzhou 510000, China; 2 Guangzhou Laboratory, Guangzhou 510000, China; 3 Guangdong Provincial Key Laboratory of Drug Non-Clinical Evaluation and Research, Guangdong Lewwin Pharmaceutical Research Institute Co., Ltd., Guangdong Engineering Research Center for Innovative Drug Evaluation and Research, Guangzhou 510000, China; 4 Division of Life Science, Center for Cancer Research, and State Key Laboratory for Molecular Neuroscience, Hong Kong University of Science and Technology, Hong Kong, China; 5 Argorna Pharmaceuticals Co., Ltd., Guangzhou 510000, China; 6 Guangzhou RiboBio Co., Ltd., Guangzhou 510000, China; 7 Technology Centre, Guangzhou Customs, Guangzhou 510000, China; 8 State Key Laboratory of Respiratory Disease, Laboratory of Computational Biomedicine, Guangzhou Institutes of Biomedicine and Health, Chinese Academy of Sciences, Guangzhou 510000, China; 9 Guangzhou Key Laboratory for Clinical Rapid Diagnosis and Early Warning of Infectious Diseases, Guangzhou 510000, China; 10 State Key Laboratory of Quality Research in Chinese Medicine, Macau University of Science and Technology, Taipa 519020, Macau (SAR), China

**Keywords:** SARS-CoV-2, vaccine, spike protein, mice, nonhuman primates

## Abstract

There is an urgent need for a broad-spectrum and protective vaccine due to the emergence and rapid spreading of more contagious SARS-CoV-2 strains. We report the development of RBMRNA-176, a pseudouridine (Ψ) nucleoside-modified mRNA-LNP vaccine encoding pre-fusion stabilized trimeric SARS-CoV-2 spike protein ectodomain, and evaluate its immunogenicity and protection against virus challenge in mice and nonhuman primates. A prime-boost immunization with RBMRNA-176 at intervals of 21 days resulted in high IgG titers (over 1:819,000 endpoint dilution) and a CD4+ Th1-biased immune response in mice. RBMRNA-176 vaccination induced pseudovirus-neutralizing antibodies with IC_50_ ranging from 1:1020 to 1:2894 against SARS-CoV-2 spike pseudotyped wild-type and variant viruses, including Alpha, Beta, Gamma, and Kappa. Moreover, significant control of viral replication and histopathology in lungs was observed in vaccinated mice. In nonhuman primates, a boost given by RBMRNA-176 on day 21 after the prime induced a persistent and sustained IgG response. RBMRNA-176 vaccination also protected macaques against upper and lower respiratory tract infection, as well as lung injury. Altogether, these findings support RBMRNA-176 as a vaccine candidate for prevention of COVID-19.

## 1. Introduction

The pandemic of severe acute respiratory syndrome coronavirus 2 (SARS-CoV-2) continues to sweep the world and has resulted in extremely high morbidity (over 4.15 billion cases) and significant mortality (over 6.49 million deaths) [1]. Intensive efforts have been made in developing vaccines that have been shown to provide significant protection against COVID-19 in patients with symptomatic to severe illness [2]. However, the development of vaccines coincided with the emergence of variants of concern (VOC), including B.1.1.7 (Alpha), B.1.351 (Beta), P.1 (Gamma), B.1.617.1 (Kappa), B.1.617.2 (Delta), B.1.1.529 (Omicron), C.37 (Lambda), and B.1.621 (Mu) [3]. Some of these variants have been reported to reduce the efficiency of currently licensed vaccines [4,5,6], which could accelerate the spread of SARS-CoV-2. Noteworthily, new VOCs are arising, and transmissibility may increase with some VOCs [7,8]. Therefore, it is imperative to develop more effective and broad-spectrum vaccines to ensure the efficient control of such a public health crisis.

To date, diverse SARS-CoV-2 vaccine candidates have been developed by using multiple platforms, such as viral vectors, protein subunits, inactivated virus, live-attenuated virus, mRNA, and DNA [9]. Of these, the mRNA technology has gained increasing interest due to various advantages, such as acceptable safety, higher efficacy, and most rapid development. The SARS-CoV-2 spike (S) protein has been thought to be one of the most essential targets for mRNA vaccine design [10]. The S protein is a trimeric transmembrane protein on the viral envelope, which comprises the S1 and S2 subunits required for host receptor binding and fusion, respectively [11]. Earlier studies on SARS-CoV and MERS-CoV demonstrated that the immune responses induced by the S protein played a significant role in antiviral immune response [12,13]. Strong binding of SARS-CoV-2 receptor-binding domain (RBD) to the ACE2 receptor resulted in a higher infection rate compared with SARS-CoV and MERS-CoV [10]. Furthermore, collective studies suggested that mutations in the S protein was associated with increased replication and transmission of SARS-CoV-2 [13]. Therefore, developing mRNA vaccines against the SARS-CoV-2 S protein could have great potential to reduce viral infection and combat its rapid spreading in the human population. Since December 2020, several mRNA vaccines encoding the SARS-CoV-2 S protein have been validated for use by the WHO, including BNT162b2 (Pfizer), mRNA-1273 (Moderna), and NVX-CoV2373 (Novavax). There are also several mRNA vaccine candidates encoding full-length S protein or the RBD under phase I/II/III clinical trials, such as CVnCoV, ARCoV, and BNT162 [14].

In the present study, we report the preclinical development of RBMRNA-176, an LNP-formulated pseudouridine (Ψ) nucleoside-modified mRNA vaccine that encodes the S ectodomain trimer with a two-proline mutation (K986P and V987P). This mutation strategy could stabilize the S protein in its prefusion conformation and prevent protease cleavage at S1/S2 and S2’ cleave sites. The wild-type signal peptide of the S protein was reported to be swapped with a signal peptide from human immunoglobulin heavy chain variable region (IGHV) for improved S post-translational translocation [15,16]. The fusion peptide was deleted to further reduce the cell fusion. The Ψ-modification could resist ribonuclease (RNase) degradation [17], reduce activation of TLRs [18] and PKR [19], and, together with optimized non-coding sequence elements, enhance in vitro translational efficacy. The pre-fusion format of the S timer is the predominate form on the virion surface and is desirable for vaccine development due to the preservation of most or all neutralization-sensitive epitopes [20,21,22,23]. We then determined the immunogenicity of RBMRNA-176 and its ability to protect mice and nonhuman primates from SARS-CoV-2 infection. Our findings indicate the potential of an effective mRNA vaccine encoding viral S protein against COVID-19.

## 2. Materials and Methods

### 2.1. Cells and Virus

African green monkey kidney epithelial (Vero E6) cells were cultured in Dulbecco’s Modified Eagle’s medium (DMEM, GIBCO, Grand Island, NY, USA) supplemented with 10% fetal bovine serum (FBS) at 37 °C. The SARS-CoV-2 virus (GISAID accession no. EPI_ISI_402124) used in the studies of mice and nonhuman primates were grown in Vero E6 cells, and viral titer was determined by plaque assay or 50% tissue culture infective dose (TCID_50_). TCID_50_ was calculated by the Reed–Muench method. All experiments using an infectious virus were performed in a biosafety level-3 (BLS-3) laboratory.

### 2.2. mRNA Synthesis and LNP Formulation

The RBMRNA-176 mRNA was synthesized in vitro by T7 polymerase-mediated transcription from a linearized DNA template, where the UTP was substituted with pseudoUTP. Capped mRNAs were generated by supplementing the transcription reactions with 10 mmol RIBO Cap1. mRNA was purified by reversed-phase high-performance liquid chromatography (RP-HPLC) as previously reported [24]. Lipid nanoparticles were prepared by microfluidic mixing using the previously described method [25]. Briefly, four lipids were dissolved in ethanol at molar ratios of 50:38.5:10:1.5 (ionizable lipid: holesterol:DSPC:DMG-PEG2000). The lipid mixture was rapidly combined with a buffer of 50 mM sodium citrate (pH 4.0) containing mRNA at a volume ratio of aqueous:ethanol using a microfluidic mixer (PNI Nanosystems, Vancouver, BC, Canada). Formulations were dialyzed against PBS (pH 7.2) in the dialysis cassettes (Thermo Scientific, Rockford, IL, USA) for at least 18 h. Formulations were diluted with PBS (pH 7.2) to reach a required concentration, and then passed through a 0.22-μm filter and stored at 4 °C until use. Formulations were analyzed for particle size, mRNA encapsulation, residues, endotoxin, and bioburdens.

### 2.3. Cryo-EM Sample Preparation

3–5 μL of the sample was dispensed on a plasma-cleaned grid in the Vitrobot chamber at 95% relative humidity and allowed to rest for 30 s. The grid was blotted with filter paper and plunged into liquid ethane cooled by liquid nitrogen. The frozen grids were then checked for visible defects and assembled into cassettes. Cryo TEM acquisition was performed at 200 kV using Falcon III and K3 cameras with DED Talos F200C (Fisher Scientific, Waltham, MA, USA).

### 2.4. Particle Size

Particle size was measured in triplicate and reported as Z-average diameter using dynamic light scattering (DLS) on a ZETASIZER (Malvern Instruments Ltd., Westborough, MA, UK). RBMRNA-176 was diluted with PBS buffer and filtered through a 0.22-μm membrane-pore diameter, shortly before acquiring its size distribution. The sample parameters used the following settings: Material Name: liposome, Cell Name: DTS0012, Dispersant Name: PBS. The temperature was maintained at 25 °C during the measurements. DLS values are the average of 10 distinct scans for 10 acquisitions on a given sample. The ZS XPLORER Software (V1.3.1.7, Malvern Panalytical, Westborough, MA, USA) was used to analyze the light scattering data.

### 2.5. Cell Transfections

10 μg of flag-tagged RBMRNA-176 mRNA or flag-tagged wild-type S mRNA was lipoplexed with 15 µL riboFECTTM mRNA transfection Reagent (C11055, Ribobio, Guangzhou, China), brought to a volume of 1 mL with Opti-MEM Reduced Serum Medium (Thermo Fisher Scientific, Waltham, MA, USA). After incubation for 5 min at room temperature, 1 mL of the mixture was added to 5 × 10^6^ HEK293T cells. The cells were cultured for 48 h at 37 °C after transfection.

### 2.6. Native Western Blot

Cells were collected and lysed in RIPA Lysis Buffer (R0030, Solarbio, Beijing, China) and centrifugated at 12,000× *g*. The supernatant was collected for native PAGE and immunoblotting. Native PAGE (6%) was performed according to the same procedure as SDS PAGE with the following modifications: the protein preparations were not boiled before electrophoresis, SDS and b-mercaptoethanol were omitted from the gels, and Tris-HCl glycine (375 mM, 0.1% SDS, pH 8.3) was used as the running buffer. After electrophoresis, the samples were transferred onto a nitrocellulose membrane using a Trans-Blot SD Semi-dry transfer cell (BioRad, Hercules, CA, USA) for 2 h at 25 V. Membranes were blocked with 5% BSA in PBS pH 7.0 and incubated overnight with anti-DDDDK Tag antibody (ab1162, 1:2500) at 4 °C. After that, the membranes were washed three times with PBS-T (0.05% Tween-20, Sigma, Shanghai, China) and incubated with HRP-conjugated Goat Anti-Rabbit (IgG) secondary antibody (ab6721, 1:4000, Abcam, Cambridge, UK) for 1 h at room temperature. Thereafter, the membranes were washed as described above, and developed with ECL reagent (P0018AS, Beyotime, Shanghai, China) using Tanon 5200 Chemiluminescent Imaging System (Tanon Science & Technology Co., Ltd., Shanghai, China).

### 2.7. Bioluminescence Imaging Studies

Luciferase mRNA was synthesized in vitro by T7 polymerase and encapsulated using the same procedure as RBMRNA-176. 6–8-week-old female BALB/c mice (*n* = 3) were intramuscularly injected with 5 μg LNP-encapsulated luciferase mRNA. 3 h after administration, animals were anesthetized with 2–3% isoflurane and injected intraperitoneally (i.p.) with luciferase substrate (0000402802, Promega, Madison, WI, USA), followed by a reaction for 5 min. Fluorescence signals were collected by In-Vivo Xtreme System (XTREME, Bruker BioSpin Corp., Billerica, MA, USA) for 120 s, and the fluorescence signals in regions of interest (ROIs) were quantified using Bruker MI SE (v7.1.2.20435, Billerica, MA, USA).

### 2.8. Vaccination and SARS-CoV-2 Challenge in Animals

In the studies of immunogenicity in the mouse model, BALB/c, C57BL/6J, and B6C3F1 mice experiments were performed in strict accordance with the requirements of Guangdong experimental animal management regulations and the Institutional Animal Care and Use Committee (IACUC Approval No.: IACUC-2020-014, 016, 017). The 6- to 8-week-old female mice were obtained from Beijing Vital River Laboratory Animal Technology Co., Ltd. and housed in an SPF Laboratory Animal Facility under a 12 h light/dark cycle, with free access to food and water. For the assessment of humoral immune responses, BALB/c mice were vaccinated with three doses of RBMRNA-176 (1, 5, or 20 μg) at an interval of 21 days. At week 2 after the second dose, the serum (*n* = 6) was collected to determine specific IgG responses to SARS-CoV-2 wild-type and Omicron S protein. At this time point, serum from mice (*n* = 3) vaccinated with 20 μg RBMRNA-176 was also selected for determination of pseudovirus neutralization against S proteins of wild-type, Alpha, Beta, Gamma, and Kappa variants. In another experiment, mice were vaccinated with 4, 20 or 50 μg RBMRNA-176 at the same interval. At week 2 after the second shot, serum (*n* = 3) was collected and subjected to pseudovirus neutralization assay using SARS-CoV-2 Omicron S protein. At week 4, the serum (*n* = 4) was also used to determine pseudovirus neutralization against wild-type S protein. In addition, neutralization of SARS-CoV-2 live virus was also determined at week 24 by using serum from mice (*n* = 4) vaccinated with 5 μg RBMRNA-176. For the measurement of Th1 immune responses, 3 species of mice were administered intramuscularly a prime-boost immunization with RBMRNA-176 (1, 5, or 20 μg) at intervals of 21 days. For negative control, PBS was used following the same experimental scheme. On day 7 after a single dose immunization, the spleens (*n* = 3) were harvested to access T cell response by cytokine ELISPOT. At week 2 after the booster shot, the serum (*n* = 3) was collected to determine antibody titer by ELISA.

We further evaluated the immunogenicity of RBMRNA-176 in rhesus macaques. Nonhuman primate study was performed in strict accordance with the guidelines set by the Animal Care and Use Committees of the Wuhan Institute of Virology (WIV), Chinese Academy of Sciences (CAS) (Approval No.: WIVA42202002-01). Briefly, 3- to 5-year-old rhesus macaques purchased from Topgene Biotechnology Co., Ltd were randomized into low-dose (0.1 mg) and high-dose (0.3 mg) RBMRNA-176 groups (*n* = 2 males and *n* = 2 females per group) and vaccinated at a 21-day prime-boost interval. From day 7 to day 119 after the first shot, the serum was collected once every 7 days to measure anti-SARS-CoV-2 S protein IgG titer.

In SARS-CoV-2 challenge studies, the K18-hACE2 transgenic mice were used and the experiments were performed in accordance with protocols approved by the Animal Care and Use Committee of Guangzhou Medical University (Acceptance number: 2018-297). Briefly, male K18-hACE2 transgenic mice, 6- to 7-weeks-old, were vaccinated with 5 or 20 μg of RBMRNA-176 following a 21-day prime-boost interval regimen. On week 4 after the second shot, mice were intranasally challenged with 5 × 10^4^ PFU of SARS-CoV-2. At 3 and 5 days post infection (dpi), the left lung and spleens were collected for histopathological examination, while the right lungs were collected to determine the viral titer using RT-qPCR assay. For macaques challenge studies, animals were divided into groups treated with placebo, low- and high-dose of RBMRNA-176 (*n* = 4 males and *n* = 4 females per group). On day 143–145 post-vaccination, macaques were intratracheally infected with 7 × 10^4^ PFU of SARS-CoV-2 in 4 mL. Nasal swabs and throat swabs were collected once daily from 1 dpi to 7 dpi. At 7 dpi, macaques were sacrificed and the lungs from 4 animals per group were collected, homogenized, and subjected to RT-qPCR detection of viral S gene expression. The lungs from the remaining 4 animals were collected for histopathologic evaluation.

### 2.9. ELISA Assay for Total IgG and IgG Subclass

For determination of the mouse total IgG, IgG1, and IgG2c, 96-well plates (JET BIOFIL, Guangzhou, China) were coated with 2 μg/mL recombinant SARS-CoV-2 spike trimer protein (SPN-C52H4, ACROBiosystems, Beijing, China) or recombinant SARS-CoV-2 Omiron spike trimer protein (SPN-C52Hz, ACROBiosystems) diluted in carbonate buffer (0.05 M, pH 9.6) and incubated at 4 °C overnight. The plates were washed with PBS-T (0.05% Tween-20) 3 times and were blocked with 1% BSA in PBS for 1–2 h at 37 °C. Next, the plates were washed and heat-inactivated serum serially diluted in PBS-T. 100 μL/well diluted serum samples were added and incubated for 1 h at 37 °C. After washing plates three times with PBS-T, HRP-conjugate Goat anti-mouse IgG (H+L) (SA00001-1, 1:1000, Proteintech, Chicago, IL, USA), HRP-conjugated Goat anti-Mouse IgG1 (A90-105P, 1:5000, Bethyl Laboratories, Inc, Montgomery, TX, USA), HRP-conjugated Goat anti-Mouse IgG2a (1:5000, Bethyl Laboratories, A90-107P) or HRP-conjugated Goat anti-Mouse IgG2c (1:5000, Bethyl Laboratories, A90-136P) was added and incubated for 1 h at 37 °C. Plates were then washed and 100 μL of TMB peroxidase substrate (PA107-02, TIANGEN, Beijing, China) was added to each well for development. After 15 min, plate development was halted by the addition of 2 mol H_2_SO_4_ and the absorbance was read at 450 nm using TECAN Infinite M200 pro (Tecan, Männedorf, Switzerland). Endpoint titers were calculated as the highest serum dilution that exceeded the cut-off values (mean ± 3SD of negative controls at the lowest dilution).

For the detection of macaque IgG, 96-well microplates were coated with 4 μg/mL of SARS-CoV-2 S protein (DRA49, Novoprotein, Suzhou, China), then rinsed with PBS-T and blocked using 1% BSA blocking buffer. Serial diluted serum samples were then added to the coated plates and incubated at room temperature for 2 h. After washing with PBS-T, the HRP-conjugated mouse anti-monkey IgG (RS2140, 1:10,000, Immunoway, Plano, TX, USA) was added and incubated at room temperature for 1 h. Plates were washed again with PBS-T, which was followed by the addition of TMB peroxidase substrate (TIANGEN, PA107-02). After incubation for 30 min, the reaction was terminated with 2 mol H_2_SO_4_. Plates were read at OD 450 nm using a BIORAD iMark microplate absorbance reader. Endpoint titers were calculated as the highest serum dilution that OD value exceeded 0.01.

### 2.10. Mouse ELISPOT Assay

Mouse ELISPOT assays were performed with Mouse IL-4 precoated ELISPOT kit (2210005, Dakewe Biotech, Shenzhen, China), Mouse IL-5 ELISpotPLUS (HRP) (3391-4HPW-2, Mabtech, Nacka Strand, Sweden), Mouse IL-2 ELISpotPLUS (HRP) (Mabtech, 3441-4HPW-2), Mouse IFN-γ precoated ELISPOT kit (2210005, Dakewe Biotech, Shenzhen, China) according to the manufacturer’s instructions. Briefly, the plates were blocked using RPMI 1640 (GIBCO) containing 10% FBS and incubated for 10 min at room temperature. Spleen lymphocytes were isolated from BALB/c mice 7 days after the first vaccination and plated at 2 × 10^5^ cells/well. Lymphocytes were stimulated with 1 μg SARS-CoV-2 Spike trimer protein (SPN-C52H4, ACROBiosystems, Beijing, China) and cultured for 20 h (3 °C, 5% CO_2_). The plates were washed 6 times with wash buffer and incubated for 1 h with biotinylated anti-mouse IL-4/IL-5/IL-2/IFN-γ antibody. The plates were washed 6 times and incubated for 1 h with Streptavidin-HRP. The final wash was followed by the addition of AEC substrate solution for 10 min. The chromagen was discarded and the plates were washed with water and dried in a dim place. Spot numbers were evaluated using ELISpotreader.

### 2.11. Flow Cytometric Analysis of T Cells and Intracellular Cytokine

BALB/c mice were vaccinated with two shots of 20 μg RBMRNA-176 at an interval of 21 days. At 7 days post-boost, splenocytes were isolated. A total of 1 × 10^6^ splenocytes (100 μL) were seeded per well into 96-well plates and incubated with 1μg SARS-CoV-2 Spike trimer protein (SPN-C52H4, ACROBiosystems, Beijing, China) or 50 ng/mL phorbol myristate acetate (PMA) and 1 μg/mL ionomycin (Dakewe Biotech, 2030421) for 2 h at 37 °C. Next, Brefeldin A Solution (420601, BioLegend, San Diego, CA, USA) was added and incubated for 4 h at 37 °C. Unstimulated cells were used as a background control, and PMA/ionomycin stimulated cells were used as a positive control. After stimulation, splenocytes were washed twice with PBS and the Zombie Green™ Fixable Viability Kits (BioLegend, 423111) was used to determine the viability of cells prior to the fixation and permeabilization. Cells were then resuspended in FC buffer (PBS + 2% FBS). Cytokine production of T cells was evaluated by surface and intracellular cytokine staining using Cyto-Fast™ Fix/Perm Buffer Set (BioLegend, 426803). PerCP/Cyanine5.5 anti-mouse CD3 antibody (BioLegend, 100218), APC anti-mouse CD8a antibody (BioLegend, 100712), and APC anti-mouse CD4 antibody (BioLegend, 100412) were used for surface staining. PE anti-mouse/human IL-5 antibody (BioLegend, 504204), PE anti-mouse IL-2 antibody (BioLegend, 503808), PE anti-mouse IFN-γ antibody (BioLegend, 505808), and PE anti-mouse IL-4 antibody (BioLegend, 504104) were used for cytokine staining. Splenocytes were analyzed on Accuri C6 (BD Biosciences, San Jose, CA, USA). 1 × 10^5^ events were collected per sample. Data were analyzed with FlowJo software. Cytokine expression in the unstimulated group was considered background and subtracted from the responses.

### 2.12. Lymphocyte Population

RBMRNA176-induced effects on the proliferation and dynamics of immune cell populations were evaluated using Accuri C6 (BD Biosciences). Briefly, 1 × 10^6^ splenocytes (100 μL) were seeded per well into 96-well plates. Following two washes with PBS, the viability of cells was determined using Zombie Green™ Fixable Viability Kits (BioLegend, 423111) prior to the fixation and permeabilization. Next, splenocytes were permeabilized using Cyto-Fast™ Fix/Perm Buffer Set (BioLegend, 426803) and stained with PerCP/Cyanine5.5 anti-mouse CD3 (BioLegend, 100218), APC anti-mouse CD8a (BioLegend,100712), APC anti-mouse CD4 (BioLegend, 100412), PE anti-mouse CD62L (BioLegend, 161203), and APC anti-mouse/human CD44 (BioLegend, 103011). Data were analyzed with FlowJo software (v10, BD Biosciences, San Jose, CA, USA).

### 2.13. Pseudoviruses Neutralization Assay

The gene sequence of SARS-CoV-2 wild-type S protein (Genebank accession NO. 43740568) or variants, including Alpha (Genebank accession NO. ON442267.1), Beta (Genebank accession NO. ON322586.1), Gamma (Genebank accession NO. ON471228.1), Kappa (Genebank accession NO. OU322235.1), BA.1 (Genebank accession NO. OM287553.1) and BA. 2 (Genebank accession NO. OM617939.1) were codon-optimized for expression in human cells. A SARS-CoV-2 pseudoviruses neutralization assay was performed using a HIV-1 lentiviral packaging system following a similar approach that was reported previously [26]. Plasmids expressing S protein and plasmids encoding luciferase-expressing lentivirus (pLV-Luc, pH 1) were co-transfected into HEK 293T cells using Lipofectamine 3000 Transfection Reagent (Thermo Fisher). Cell suspensions enriched with the pseudotype virus were harvested after 48 h and TCID_50_ was measured. Pseudoviruses were stored at −80 °C.

Serial 3-fold diluted inactivated serum, starting at 1:10, were incubated with 300 TCID_50_ of the pseudovirus for 1 h at 37 °C in 96-well plates. DMEM was used as negative control and lentiviral SARS-CoV-2 pseudoviruses were used as positive control. After incubation, 4 × 10^4^ 293T-ACE2-p2A-mTagBFP2 cells (a cell line stably expressing ACE-2) were added and incubated for 48 h. Following this, cells were lysed, and luciferase signal was measured using GloMax^®^ 96 Microplate Luminometer (Promega). The inhibition curve was determined by non-linear regression, i.e., log (inhibitor) vs. normalized response (variable slope) using GraphPad Prism 8.0 (GraphPad Software, Inc., San Diego, CA, USA) and IC_50_ titers were calculated using the Reed–Muench method.

### 2.14. Live Virus Neutralization Assay

Serum samples collected from immunized mice were inactivated at 56 °C for 30 min and serially diluted with DMEM medium (GIBCO) in two-fold steps. The diluted serums were mixed with 100 TCID_50_ SARS-CoV-2 live virus in 96-well plates at a ratio of 1:1 (*v*/*v*) and incubated at 37 °C for 1 h. Next, virus/serum mixtures were added to Vero-E6 cells monolayers in quadruplicate in 96-well plates and the plates were incubated for 3–5 days at 37 °C in a 5% CO_2_ incubator. The cytopathic effect (CPE) of each well was recorded under microscopes, and the 50% neutralization titer (NT_50_) was calculated.

### 2.15. RT-qPCR Assay

The total RNA from the supernatants of nasal swab, throat swab, and lung homogenization was extracted by using QIAamp Viral RNA mini-Kit (Qiagen, Hilden, Germany), and reverse transcription was performed by using the PrimeScript RT Master Mix kit (Takara Bio, Shiga, Japan). RT-PCR was then carried out with primers specific to the SARS-CoV-2 Orf1ab-N gene by using the SARS-CoV-2 Orf1ab-N Probe qRT-PCR Kit (15-81930, Tiandz, Inc., Beijing, China). RT-PCR was performed at the reaction conditions of 50 °C for 30 min, 94 °C for 10 min, followed by 40 cycles of 94 °C for 15 s, and 60 °C for 1 min. The resulting data were further analyzed by using the detection platform StepOne Real-Time PCR System (Applied Biosystems Co., Ltd., Foster City, CA, USA). Standard curves were generated by using serial ten-fold dilutions of control substance (from 1 × 10^8^ copies/μL − 1 × 10^3^ copies/μL). The viral load was expressed as copies/mL.

### 2.16. Histopathology

Mice lungs and spleens, as well as macaque lungs, were fixed in 4% paraformaldehyde and cut into 4 µm-thick paraffin sections, which was further stained with hematoxylin-eosin (HE) and captured by light microscopy (ECLIPSE NI-U, Nikon, Tokyo, Japan). Semiquantitative assessment of SARS-CoV-2-induced inflammation in 6 lobes of lungs from macaques was performed regarding the extent of pink-stained exudation in alveolar septum, inflammatory cell infiltration in perivascular and alveolar space, and the presence of interstitial pneumonia. The histopathology was scored as: 0, normal; 1, slight; 2, mild; 3, moderate; 4, severe.

### 2.17. Statistical Analysis

Statistical analysis of the virologic and immunologic data was performed using GraphPad Prism 8 software (GraphPad Software, Inc.). Values of *p* < 0.05 were considered as significant. The used statistical tests were the two-way ANOVA test with Tukey’s multiple comparisons test and non-linear regression model.

## 3. Results

### 3.1. Generation of RBMRNA-176

**Figure 1:**
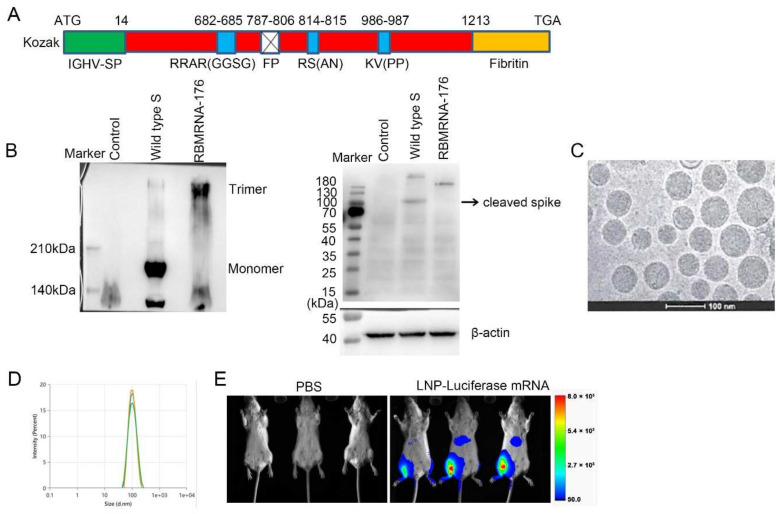
Construction of RBMRNA-176 (**A**). Native PAGE and Western blot analysis for the expression of wild-type S protein or S ectodomain trimer in transfected cell lysates (left). SDS-PAGE and Western blot analysis for S protein cleavage (right) (**B**). Cryo-TEM of RBMRNA-176 (**C**). Particle size distribution of RBMRNA-176 was measured in triplicate using dynamic light scattering (DLS) on a Malvern ZETASIZER (**D**). Biodistribution of LNP-Luciferase 357 mRNA (**E**). BALB/c mice were administrated intramuscularly (i.m.) with 5 μg LNP-packaged luciferase mRNA and bioluminescence was measured 3 h post injection in an In-Vivo Xtreme System.

In the present study, we designed a prefusion SARS-CoV-2 spike (S) RNA vaccine (Figure 1A), RBMRNA-176, which encoded the expression of virus S ectodomain with an N-terminal IGVH signal peptide for improved S post-translational translocation and a C-terminal T4 fibritin trimerization domain for trimer formation. The fusion peptide was deleted to further reduce the cell fusion. The 2 prolines (K986P/V987P) mutation stabilized the S Protein in prefusion conformation and amino acid substitutions prevented protease cleavage at an S1/S2 protease cleavage site and an S2’ protease cleave site. Native PAGE and immunoblotting results (Figure 1B) showed a view of the trimeric S proteins in the RBMRNA-176 mRNA transfected-cell culture lysis. The morphologies of RBMRNA-176 particles were analyzed by Cryo-transmission electron microscopy (Cryo-TEM) (Figure 1C), and the image showed that RBMRNA-176 exhibited homogenous morphologies of solid spheres. Particle size was measured using a ZETASIZER (Figure 1D), showing an average particle size of ~99 nm (Figure 1D) with >95% encapsulation. Next, we evaluated the in vivo delivery capability of RBMRNA-176 in mice (Figure 1E). BALB/c mice were administrated intramuscularly (i.m.) with LNP-packaged luciferase mRNA that was prepared using the same procedure as RBMRNA-176. Bioluminescence was measured 3 h later in an In-Vivo Xtreme System. Robust luciferase expression at the injection site (muscle) was detected in LNP-luciferase mRNA group with a week bioluminescent signal observed in the liver. These data indicated that the RBMRNA-176 was dominantly restricted to the injection muscle, which avoids hepatic lipid accumulation. 

### 3.2. Immunogenicity of RBMRNA-176 in Mice

**Figure 2:**
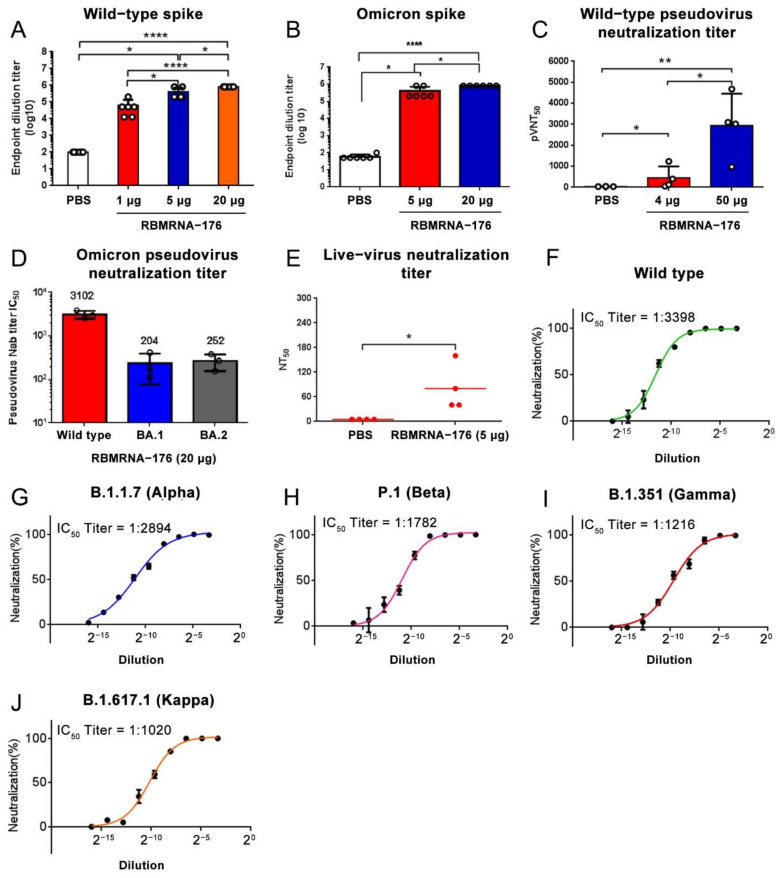
Humoral immune responses in RBMRNA-176-vaccinated mice. BALB/c mice were vaccinated with three doses of RBMRNA-176 (1, 5, or 20 μg) at an interval of 21 days. At week 2 after the second dose, the serum was collected to determinate specific IgG responses to SARS-CoV-2 wild-type (**A**) and Omicron (**B**) S proteins. At this time point, the pseudovirus neutralization of S proteins of the wild-type, Alpha, Beta, Gamma, and Kappa variants was also measured using serum from mice vaccinated with 20 μg RBMRNA-176 (**F**–**J**). Mice were vaccinated with 4 or 50 μg RBMRNA-176 at the same interval. The pseudovirus neutralization of SARS-CoV-2 wild-type (**C**) and Omicron (**D**) S protein by mouse serum was determined at weeks 4 and 2, respectively. At week 24 after vaccination with 5 μg RBMRNA-176, serum samples of mice were assayed for neutralization of SARS-CoV-2 live virus (GISAID accession no. EPI_ISI_402124) (**E**). * *p* < 0.05, ** *p* < 0.01, **** *p* < 0.0001.

BALB/c Mice were administered intramuscularly with a prime-boost immunization with RBMRNA-176 (1,4, 5, 20 or 50 μg) at intervals of 21 days. Two weeks after boost immunization, wild-type and Omicron spike-specific IgG titer in BALB/c increased in a dose-dependent manner (range, 1.28 × 10^4^ to 8.19 × 10^5^ log dilution) (Figure 2A,B). The neutralizing capacity of antibodies was assessed with a pseudovirus neutralization assay and a live virus plaque reduction neutralization test (PRNT). Pseudovirus neutralization assays showed that two shots of 50 μg RBMRNA-176 induced a higher level of neutralizing antibodies against the wild type (IC_50_ titer mean value 2941) than 4 μg RBMRNA-176 (IC_50_ titer mean value 453) (Figure 2C), and that RBMRNA-176 vaccination resulted in 15- and 12-fold reduction in antibody responses to BA.1 and BA.2, respectively, compared to the wild type (Figure 2D). Live virus plaque reduction neutralization assays demonstrated that all the 5 μg RBMRNA-176-vaccinated mice developed detectable neutralizing antibodies even after 6 months (Figure 2E). These results indicate that two-dose immunization with RBMRNA-176 induced robust antibody responses in mice. In addition, serum collected from BALB/c Mice vaccinated with 20 μg of RBMRNA-176 neutralized not only wild-type SARS-CoV-2 with IC_50_ of 1:3398, but also multiple variants such as Alpha, Beta, Gamma, and Kappa, with IC_50_ ranging from 1:1020 to 1:2894 (Figure 2F–J). These results indicate that RBMRNA-176 has the potential to induce a broad-spectrum anti-viral immune response against SARS-CoV-2 variants in mice.

**Figure 3:**
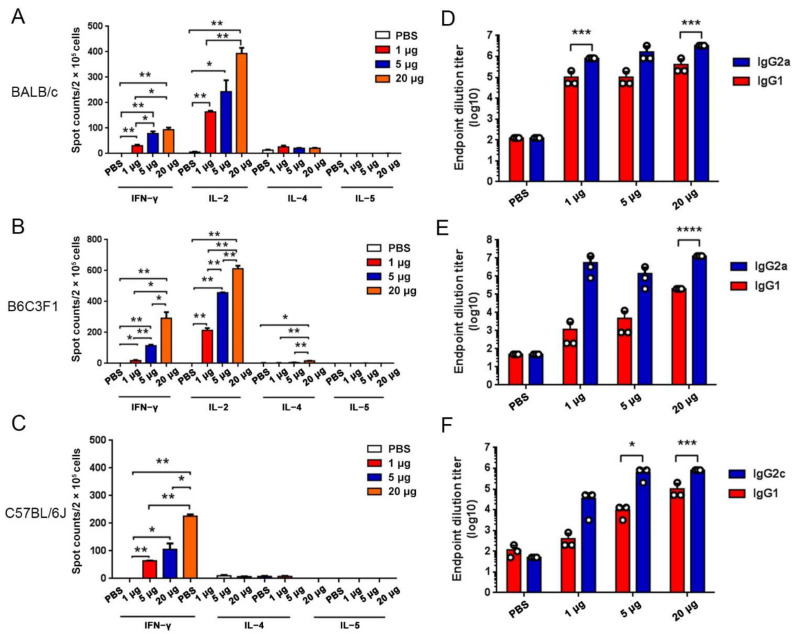
RBMRNA-176 immunization induced Th1-skewed immune responses in mice. BALB/c, B6C3F1 and C57BL/6J mice were vaccinated with two shots of RBMRNA-176 (1, 5, or 20 μg) at an interval of 21 days. On day 7 after the first shot, mice splenocytes were prepared for IFN-γ, IL-2, IL-4, and IL-5 ELISPOT assay (**A**–**C**), and at week 2 after the second shot the serum was collected to determinate IgG1 and IgG2c/IgG2a endpoint titers (**D**–**F**). * *p* < 0.05, ** *p* < 0.01, *** *p* < 0.001, **** *p* < 0.0001.

Vaccination with RBMRNA-176 induced significant IFNγ and IL-2 ELISPOT responses but minimal or no IL-4 and IL-5 responses in three species of mice (Figure 3A–C). IgG2c/IgG2a response was stronger than IgG1 in multiple species of mice (Figure 3D–F). These results suggest an induction of Th1-biased immune responses following RBMRNA-176 vaccination in mice.

**Figure 4:**
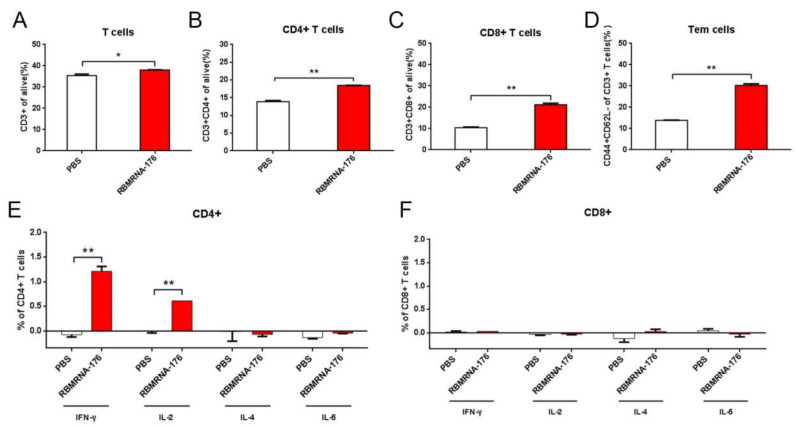
Assessment of RBMRNA-176-induced T cell responses and effects on proliferation of immune cell populations in the spleen. BALB/c mice were vaccinated with two shots of 20 μg RBMRNA-176 at an interval of 21 days. 7 days post-boost, splenocytes were isolated from 3 mice per group, and lymphocyte subset percentages (CD3+, CD4+, CD8+ and Tem) were measured (**A**–**D**). Splenocytes were re-stimulated with 1 μg SARS-CoV-2 Spike trimer protein. After 6 h, CD4+ T cells (**E**) or CD8+ T cells (**F**) expressing IFN-γ, IL-2, IL-4, and IL-5 in response to the S Protein were detected by intracellular cytokine staining (ICS). * *p* < 0.05, ** *p* < 0.01.

We further studied RBMRNA-176-induced SARS-CoV-2-specific T cell immune response and its effects on the proliferation of immune cell populations. Flow cytometry results showed a significant increase in CD3+, CD4+, CD8+ and effector memory T cells (Tem) in the spleens of the RBMRNA-176 vaccinated group compared with the PBS control group (Figure 4A–D). Consisted with the ELISPOT results, the secretion of IFN-γ and IL-2 in CD4+ T cells from the RBMRNA-176-immunized mice was significantly higher than IL-4 and IL-5 (Figure 4E), indicating Th1 responses were stronger than Th2 responses. In contrast, CD8+ T cell responses were low to undetectable after RBMRNA-176 vaccination (Figure 4F). Our results demonstrated that RBMRNA-176 successfully induced a systemic CD4+ Th1-biased immune response.

### 3.3. Protective Efficacy of RBMRNA-176 against SARS-CoV-2 Challenge in Mice

**Figure 5:**
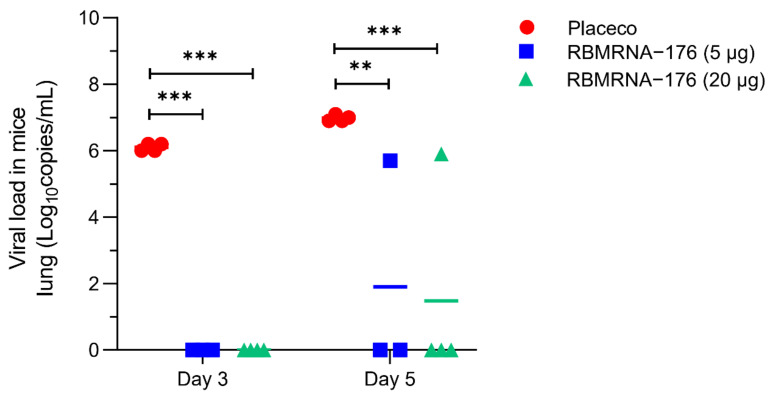
Viral load in lungs of SARS-CoV-2-infected mice vaccinated with RBMRNA-176. K18-hACE2-transgenic mice were vaccinated with 20 μg RBMRNA-176 following a 21-day prime-boost interval regimen. At week 4, mice were intranasally infected with 5 × 10^4^ PFU of SARS-CoV-2. Viral titer (*n* = 4) was determined by RT-qPCR detection of viral s gene copies in homogenized supernatants of lung tissues at 3 and 5 dpi. ** *p* < 0.01, *** *p* < 0.001, compared with placebo-treated control without RBMRNA-176 vaccination.

To evaluate the protective efficacy of RBMRNA-176, K18-hACE2-transgenic mice were intranasally challenged with SARS-CoV-2 on day 28 after receiving a booster shot. Vaccination with RBMRNA-176 at a dose of 5 or 20 μg neutralized viral replication to an undetected level at 3 dpi (*p* < 0.001) (Figure 5). At 5 dpi, vaccination with RBMRNA-176 also significantly reduced viral titer (*p* < 0.01, *p* < 0.001), but virus replication was still evident in one of four mice (Figure 5).

**Figure 6:**
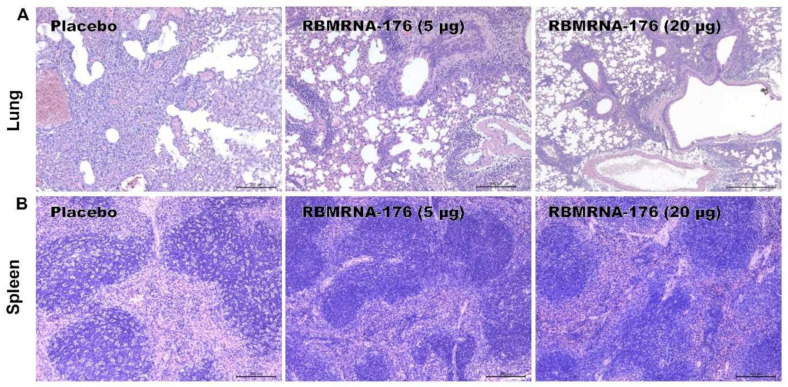
Histopathological changes in lungs and spleens in mice challenged with SARS-CoV-2. RBMRNA-176-vaccinated mice were intranasally challenged with 5 × 10^4^ PFU of SARS-CoV-2. At 5 dpi, lungs (**A**) and spleens (**B**) were collected for hematoxylin and eosin (H&E) staining and examination of histopathologic changes (*n* = 4; original magnification: 100×; scale bar: 200 μm).

By histopathology, placebo-treated mice displayed multiple signs of histological damage in lungs, including congestion, hemorrhage, alveolar lumen exudates, and alveolar expansion (Figure 6A). Additionally, these animals exhibited significant lymphocyte apoptosis in spleens, as indicated by a starry-sky pattern (Figure 6B). In contrast, mice vaccinated with a high dose of RBMRNA-176 had unapparent lesions in their lungs or spleens.

### 3.4. Immunogenicity and Protection against SARS-CoV-2 Challenge following RBMRNA-176 Immunization in Rhesus Macaques

**Figure 7:**
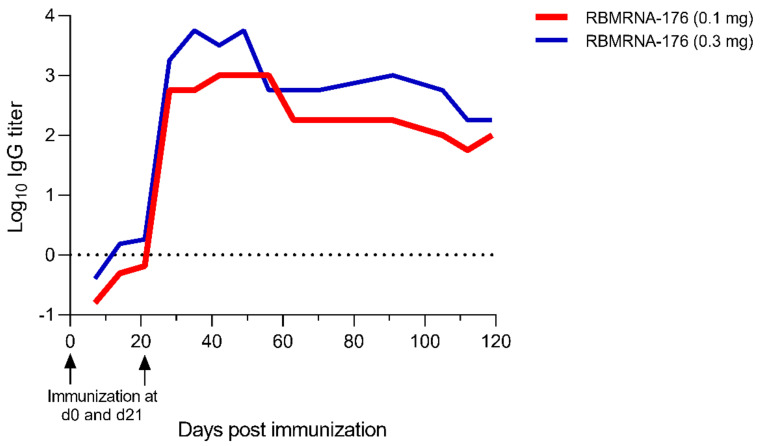
Humoral immune response in RBMRNA-176-vaccinated rhesus macaques. Rhesus macaques were vaccinated with 0.1 or 0.3 mg RBMRNA-176 at a 21-day prime-boost interval. From day 7 to day 119 after the first shot, the serum was collected daily and subjected to IgG ELISA assay.

We then investigated the immunogenicity of RBMRNA-176 in rhesus macaques that received a prime on day 0 and a booster on day 21. It was shown that vaccination of RBMRNA-176 induced persistent IgG responses in a dose-dependent manner and that the IgG titer remarkably increased on day 7 and peaked on day 28 (7.8 × 10^3^ log IgG, high dose group), maintaining constantly to day 119 (Figure 7). 

**Figure 8:**
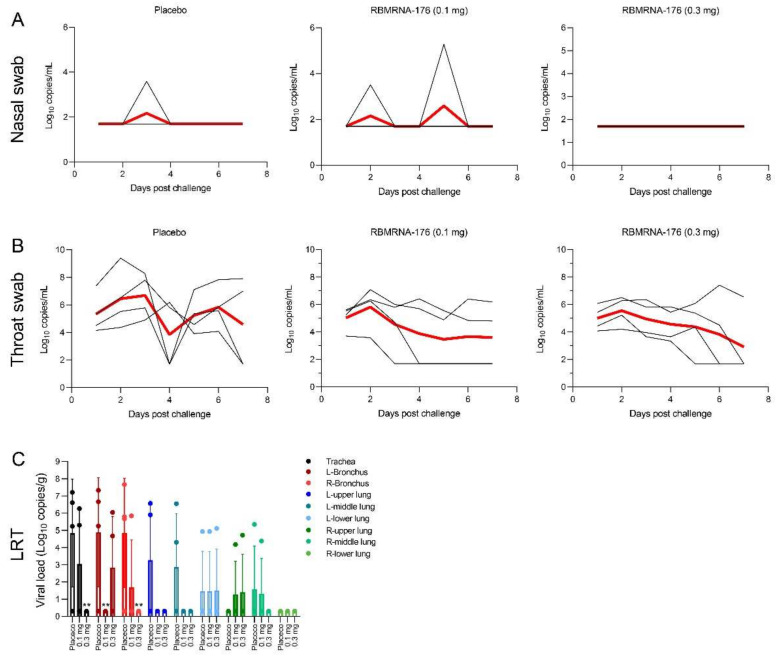
Viral load in respiratory tract tissues of SARS-CoV-2-infected rhesus macaques with RBMRNA-176 vaccination. RBMRNA-176-vaccinated rhesus macaques were intranasally challenged with 10^5^ TCID_50_ of SARS-CoV-2. Log s gene copies/mL (*n* = 4, limit of detection 200 copies/mL) were determined by using nasal swabs (**A**) and throat swabs (**B**) collected daily from 1 to 7 dpi. Red lines presented the median viral loads. (**C**) Viral titer (*n* = 4) in the lower respiratory tract (LRT), including trachea, bronchus, and lungs, was assessed by TCID_50_ assay at 7 dpi. Red and black lines present median and individual viral load, respectively. ** *p* < 0.01, compared with placebo-treated control without RBMRNA-176 vaccination.

At day 145 after the first dose of RBMRNA-176, macaques were challenged with 10^5^ PFU of SARS-CoV-2 and shed virus to average peaks of 2.17 (range 1.70–3.59) and 6.69 (range 4.90–7.80) log S gene copies in nasal swab and throat swab, respectively (Figure 8A,B). In contrast, treatment with 0.3 mg of RBMRNA-176 reduced virus copies in nasal swabs to an undetectable level (Figure 8A). Macaques that were treated with 0.1 and 0.3 mg RBMRNA-176 had 0.88- and 1.13-fold reduction in peak virus titer in throat swabs, respectively (Figure 8B). Regarding the anti-viral efficacy in respiratory tissues, RBMRNA-176 administration resulted in a reduction (0.1 mg, median, 3.18-fold; 0.3 mg, median, 3.71-fold, *p* < 0.01) in the amount of virus in the tracheal and bronchus areas (Figure 8C). Similarly, macaques receiving RBMRNA-176 had a 0.79- to 0.95-fold lower amount of virus in the lungs as compared with placebo-treated controls (Figure 8C). Notably, most of the animals immunized by 0.3 mg RBMRNA-176 had no detectable virus in the lungs (Figure 8C).

**Figure 9:**
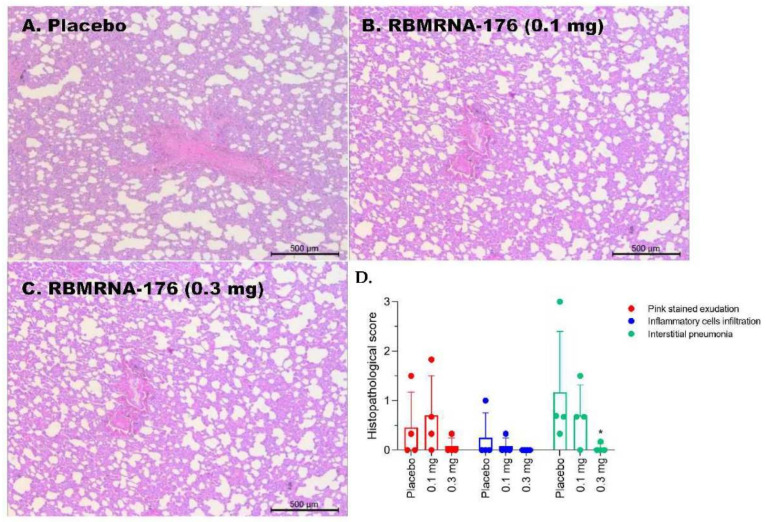
Histopathologic changes in lungs from RBMRNA-176-vaccinated rhesus macaques challenged with SARS-CoV-2. Pathological changes in the hematoxylin and eosin (H&E) staining of paraffin-sectioned lung tissues (original magnification: 50×; scale bar: 500 μm) (**A**–**C**). Comparison of histopathological scores between vaccinated and normal macaques (*n* = 4) (**D**). * *p* < 0.05, compared with placebo-treated control without RBMRNA-176 vaccination.

Next, the effect of RBMRNA-176 vaccination on histopathological changes in the lungs of SARS-CoV-2-infected macaques was evaluated. In placebo-treated animals, there was mild-to-severe interstitial pneumonia and mild-to-moderate pink-stained exudation in the alveolar septum (Figure 9A). Inflammatory cell infiltration in perivascular and alveolar space was also present in one of the four animals. Being vaccinated with a high dose of RBMRNA-176 resulted in a significant reduction in the severity of parenchymal lung inflammation (*p* < 0.05) (Figure 9B–D).

## 4. Discussion

In the present study, we designed a prefusion SARS-CoV-2 S RNA vaccine, RBMRNA-176, that expressed S ectodomain and stabilized the prefusion S trimer by introducing two-proline mutation (K986P and V987P). The two-proline mutation plays a key role in inducing neutralizing antibodies and has been adopted in the development of other licensed mRNA vaccines, such as Moderna mRNA-1273, Pfizer BNT162b and Novavax NVX-CoV2373 [11,27,28]. In addition, studies showed that the Ψ nucleoside-modification resulted in a 10-fold increase in translation, and this processing did not activate pathogen-associated molecular pattern sensing machinery, thereby reducing redundant inflammation and, hence, vaccine-related adverse effects [29,30]. Such modification could improve the translatability and safety of RBMRNA-176 vaccine. A previous study by Y.-M. Kwon et al. reported that RSV F glycoprotein with fusion peptide deletion and cleavage site mutation induced higher levels of IgG antibodies and RSV-neutralizing activity titers, as well as efficient lung virus clearance [31]. In contrast, recent studies showed that the fusion peptide was a candidate epitope site for pan-coronavirus-neutralizing antibodies [32,33]. It has also been suggested that mAbs with broader epitope coverage outside of the RBD exhibited higher protection from MERS-CoV and SARS-CoV infection [34,35]. Therefore, further efforts should be made to develop a fusion peptide-based vaccine with increased virus-neutralizing activity compared to RBMRNA-176.

We showed that RBMRNA-176 vaccination resulted in Th1-skewed response as determined by the production of Th-1 cytokines IFNγ and IL-2 by total CD4 T cells, which was similar to previous studies using Pfizer BNT162b2 and Moderna mRNA-1273 vaccines [28,36]. The Th1-skewed response is critical for viral control and is important for the development of SARS-CoV-2 vaccines [37]. In contrast, Th2 polarization may be related to vaccine-associated enhanced respiratory disease (VAERD) [37]. Thus, the Th1 bias induced by RBMRNA-176 vaccination may reduce the risk of unfavorable effects triggered by Th2 functional polarization. In addition, RBMRNA-176 elicited a significantly stronger IgG2a response than IgG1, and the ratio of IgG2a/IgG1 has been thought to reflect the balance of Th1- and Th2-response. It has also been shown that higher IgG2a titer contributes to macrophage-induced cell-mediated cytotoxicity (ADCC) effect and opsonophagocytosis, hence facilitating virus clearance [38,39]. The IgG response elicited by RBMRNA-176 vaccination appeared comparable to that induced by Pfizer BNT162b2 in mice, as determined by pseudovirus neutralization assays [28]. The dynamic IgG response was also comparable across nonhuman primates vaccinated with RBMRNA-176 or Pfizer BNT162b2 at a dosage of 100 μg [28]. Following vaccination with RBMRNA-176, the mice had increased IgG and mAb titers and a robust CD4 T cell response, but a negligible-to-undetectable CD8 T cell response in mice. Increased CD4 T cell response could possibly result in high IgG and mAb titer, whereas CD8 T cells have not been shown to be required for mAb titer or effective protection against SARS-CoV-2 infection via vaccination [37,40]. Therefore, the pronounced humoral immune response evoked by RBMRNA-176 is most likely attributable to CD4 T cell activation rather than CD8 T cells, which is consistent with the overall effect of mRNA-1273 and BNT162b2 against SARS-CoV-2 [41]. However, the limitations of our work on RBMRNA-176 immunogenicity are that we did not determine cellular immune responses in macaques, evaluate the neutralization of SARS-CoV-2 wild type and variants with macaque serum, or compare the immunogenicity of wild-type S protein with RBMRNA-176.

According to our findings, RBMRNA-176 induces high levels of neutralizing antibodies against the wild-type virus and a range of VOCs, including Alpha, Beta, Gamma, and Kappa variants, although this vaccine exhibits slightly reduced (0.85- to 3.33-fold) neutralizing potency to VOCs compared to the wild type. Although the Omicron variant could escape from antibody neutralization induced by current vaccines, T cell epitopes were confirmed conserved in this variant and memory T cells may partially respond to its S protein [42]. An mRNA vaccine also provided a long-term immune protection against SARS-CoV-2 in animal models [8]. However, Omicron was found to be the most resistant VOC to neutralization based on the results obtained from mRNA-vaccinated individuals [43]. Shan-Lu Liu et al. also demonstrated that immunity waned by 6 months after vaccination in human population and that minimal neutralizing antibody responses to the Omicron variant were observed at this time point [44]. In addition, recent studies have shown that COVID-19 vaccines based on previous strains provided less protection against the Omicron variants, including BA.4 and BA.5, due to the escape from antibody response [45,46,47]. L452R, F486V and F486V mutations contributed to BA.4/5 evasion of neutralizing antibodies [45,47]. K417N and E484A were two mutations in the RBD of Omicron that were thought to be the driving factors in vaccine breakthroughs [46]. We have determined the cross-neutralization activity induced by RBMRNA-176 vaccination against BA.1 and BA.2 pseudoviruses and found a 15- and 12-fold reduction, respectively, as compared to the wild-type virus. These results indicate that Omicron BA.1 and BA.2 have substantial but incomplete escape of RBMRNA-176-induced neutralization; nevertheless, RBMRNA-176 induced-neutralization against these variants needs to be confirmed using live viruses.

The K18-hACE2 mice and nonhuman primates allowed us to study the protective effect of vaccines on respiratory disease resembling severe COVID-19. We showed that RBMRNA-176 vaccination could significantly neutralize an infectious virus in the lungs of mice as well as in the respiratory tract and lungs of macaques. Following the same vaccination strategy as RBMRNA-176, 5 μg BNT162b2 reduced viral titer to undetectable levels in all mice at 5 dpi [48]; however, one of four mice immunized with RBMRNA-176 at this dose still showed virus growth at this time point. Similarly, two shots of Novavax NVX-CoV2373, Moderna mRNA-1273 or Pfizer BNT162b2 appeared to be more effective than RBMRNA-176 in neutralizing the virus in the lungs of mice and nonhuman primates [11,28,49]. Furthermore, the in vivo protection of RBMRNA-176 vaccination against SARS-CoV-2 variant infection warrants further investigation.

## 5. Conclusions

Taken together, our study provides insight into the broad neutralization and in vivo protection against SARS-CoV-2 infection with RBMRNA-176 vaccination. Due to RBMRNA-176 evasion by Omicron and the relatively high vaccination dose, it will be necessary to develop a vaccine of the next generation.

## Data Availability

Data can be obtained by contacting the corresponding authors.
